# A biomechanical test model for evaluating osseous and osteochondral tissue adhesives

**DOI:** 10.1186/s42490-019-0011-2

**Published:** 2019-05-07

**Authors:** Philip Procter, Michael Pujari-Palmer, Gry Hulsart-Billström, David Wenner, Gerard Insley, Sune Larsson, Håkan Engqvist

**Affiliations:** 1 Division of Applied Materials Science, Department of Engineering Sciences, Uppsala University, Box 523, 75120 Uppsala, Sweden; 2 GPBio Ltd, Rathkeale, Ireland; 3 Department Surgical Sciences, Orthopaedics, Uppsala University Hospital, 75185 Uppsala, Sweden

**Keywords:** Biomechanical model, Bone adhesive, Fracture repair, Orthobiologics, Phosphoserine, Calcium phosphate cements, Tissue adhesive

## Abstract

**Background:**

Currently there are no standard models with which to evaluate the biomechanical performance of calcified tissue adhesives, in vivo*.* We present, herein, a pre-clinical murine distal femoral bone model for evaluating tissue adhesives intended for use in both osseous and osteochondral tissue reconstruction.

**Results:**

Cylindrical cores (diameter (Ø) 2 mm (mm) × 2 mm depth), containing both cancellous and cortical bone, were fractured out from the distal femur and then reattached using one of two tissue adhesives. The adhesiveness of fibrin glue (Tisseel^tm^), and a novel, biocompatible, calcium phosphate-based tissue adhesive (OsStic^tm^) were evaluated by pullout testing, in which glued cores were extracted and the peak force at failure recorded. The results show that Tisseel weakly bonded the metaphyseal bone cores, while OsStic produced > 30-fold higher mean peak forces at failure (7.64 Newtons (N) vs. 0.21 N). The failure modes were consistently disparate, with Tisseel failing gradually, while OsStic failed abruptly, as would be expected with a calcium-based material. Imaging of the bone/adhesive interface with microcomputed tomography revealed that, for OsStic, failure occurred more often within cancellous bone (75% of tested samples) rather than at the adhesive interface.

**Conclusions:**

Despite the challenges associated with biomechanical testing in small rodent models the preclinical ex-vivo test model presented herein is both sensitive and accurate. It enabled differences in tissue adhesive strength to be quantified even for very small osseous fragments (<Ø4mm). Importantly, this model can easily be scaled to larger animals and adapted to fracture fragment fixation in human bone. The present model is also compatible with other long-term in vivo evaluation methods (i.e. in vivo imaging, histological analysis, etc.).

## Background

Each year up to 3% of the global population experience a fractured bone, with the lifetime fracture incidence exceeding 1 in 3 in elderly patients [1–3]. Up to 10% of traumatic fractures require surgical intervention, and a significant subset of these fractures are close enough to joints to produce osteochondral fragments that require fixation, or alignment [4]. Osteochondral bone fragments can impede healing, and malunion with an intra-articular or extra-articular deformity is a frequent complication [5, 6]. A standard treatment for stabilisation of fractured bone is fixation with metal implant hardware (i.e. plates, screws, nails etc.). While metal hardware has dramatically improved outcomes of fracture treatment, limitations remain, including device failure (such as screw stripping and loosening) in cases where the surrounding bone is weak, secondary fractures originating from stress concentrations and pilot holes, and bone resorption resulting from stress shielding [7, 8]. An effective bone adhesive would have a number of advantages, either as a complement to, or even as a replacement for, standard metallic implants. Tissue adhesives can directly bond to weak or osteoporotic bone surfaces, facilitate realignment and fixation of tissue fragments and reduce surgical time [9], facilitate early load bearing, and avoid detrimental stress related effects [8, 10–12].

A number of adhesives have been proposed for fracture repair, including acrylate-based glue [12], collagen or fibrin glues [13], click chemistry-based glues [14], and, more recently, adhesive ceramics [15–17]. While naturally derived adhesives, such as fibrin glue (Tisseel), are cyto- and biocompatible and may retain biologic elements that enhance healing, their bond strengths (5–100 KiloPascal (KPa) are relatively poor compared to the properties of cancellous and cortical bone [11, 18]. On the other hand, synthetic adhesives, such as cyanoacrylates, produce much greater bond strengths yet perform poorly under wet-field conditions (1 MegaPascal (MPa) dry vs. 0.1 MPa wet), and few synthetic adhesives have been optimized specifically for bone [14, 18–20]. Synthetic adhesives also degrade slowly, or often do not degrade at all, which can impede tissue remodeling and prevent complete healing of the treated site. An example of a synthetic adhesive approach is a thiol-ene chemistry fracture repair model in rats, which can attain shear strengths up to 9 MPa ex vivo [14]. Another synthetic bone cement branded Kryptonite was Food and Drug Administration (FDA) approved for cranioplasty in 2009 [21, 22]. Kryptonite was extremely tacky during curing, resulting in such effective bonding to bone tissue that many surgeons used it “off label” for its adhesiveness rather than its approved use as a void filler. The preclinical data submitted to the regulating authorities in Canada gave rise to safety concerns [23]. This product was abruptly removed from all markets following an FDA class 2 worldwide voluntary recall notice in April 2012.

There are many requirements, which a bone tissue adhesive must meet to be considered safe and effective. These are detailed in several comprehensive review papers [7, 9, 24], the most obvious requirements being biocompatibility, efficacy in wet, proteinaceous, and fatty environments, and to recreate tissue bond strength without impeding the native tissue healing process. Despite a number of adhesives that have been proposed for bone tissue reconstruction [7, 18, 22], the intense demands for proving both safety and efficacy explain why it remains an unmet clinical need. At the present time the United States food and drug administration (FDA) has not granted approval to market a bone adhesive for internal use in Humans [9]. In Europe, following the recent medical device directive changes (Medical Device Reporting MDR 2017/745) [25], a bone adhesive is automatically class 3 and so human clinical data collection is considered mandatory prior to seeking regulatory approval for clinical use.

Prior to establishing safety and efficacy in human trials, a new bone adhesive must first be characterized in pre-clinical models. At present, however, there are no standard pre-clinical models that would enable evaluation of the effectiveness of a bone adhesive [9]. The preclinical effectiveness of bone adhesives has been evaluated either by simple mechanical testing of sectioned tissue, ex vivo*,* or by more complex in vivo and ex vivo biomechanical testing of whole tissues. The preclinical efficacy of calcified tissue adhesives has been evaluated using mechanical test models that include: shear force, on cubed bone tissue (i.e. cubes cut from fresh bone, and tested in shear by gluing the cube surfaces together before applying a shear force [12, 17, 26]; tensile loading [8, 10]; or push-out, which is a commonly used test for adhesive bonding in dental applications [27]. In these studies the mechanical testing regimen did not reflect physiological loading, which is complex and may include bending, compression, shear and torsion [28, 29].

Calcified tissue adhesives have also been evaluated in whole tissues, ex vivo and in vivo, by: tensile fracture testing in canine knees, where fibrin glue was compared to Kirschner wire fixation in vivo [13]; peel testing of human periosteum fixated with a glutaraldehyde modified serum based adhesive, compared to untreated periosteum [30]; bending fracture testing of a gelatin based adhesive, compared to untreated fractures, in a murine tibial fracture model, in vivo [31]; multi-modal testing of a polymeric adhesive in a porcine femoral fracture model, ex vivo [32]; three-point bending testing in a porcine metacarpal fracture model, compared with Kirschner wire fixation, ex vivo, and peel testing on rat femoral tissue, in vivo, using a novel click-chemistry adhesive [14].

These prior studies, in part, suggest that measurable forces can be obtained from bone core models, similar to the model in the present study, and that it may be possible for adhesives to be compared and evaluated ex vivo*.* However, most of the reviewed studies do not have intra- or inter-study controls (i.e. fibrin glue, or cyanoacrylate), by which their study model and results, can be compared. As part of the development process underlying the present model, we have considered the following as minimal requirements for a useful preclinical test model:

- Inclusion of a commercially produced, clinical grade, control adhesive material Tisseel^tm^ fibrin glue was selected as although it is not approved for bone adhesion it has been most frequently used for this purpose in animal studies
- Quantitative, rather than qualitative data
- Reproducible and sensitive enough to distinguish the performance range of each adhesive ex vivo
- Subject to validation in subsequent animal studies, has the potential to be predictive of the performance of adhesive(s) in vivo
- Assesses the adhesion strength of cancellous bone, with an overlying cortical bone shell, to a similar tissue surface combination
- Preparation and delivery, of adhesive, to surgical sites should be easily done and translates directly to actual clinical surgical scenarios

In this study a pre-clinical model to evaluate the bond strength of bone adhesives, for reconstructing fragments of metaphyseal bone, is described. The aim of this study was to determine whether a metaphyseal model of bone reconstruction could distinguish between the adhesion strength of two tissue adhesives in a murine rat model, accurately and reproducibly, despite the limitations inherent to a small animal osseous defect model (i.e. miniscule defect size, large variability in bone composition, difficulty in designing a reconstructive approach that mirrors the clinical situation in humans, etc.). The present study was limited in that only two adhesives were tested, and that these adhesives differed substantially in both composition and material properties. OsStic sets/cures into a rigid, stiff adhesive. Calcium phosphate cement would be the most appropriate comparison material, based upon the material properties. However, calcium phosphates are not adhesive and would not be expected to produce measurable force in the current ex vivo preclinical model. Instead, fibrin glue (TISSEEL) was selected as the second tested material because, although TISSEEL is a soft, elastic material, it produces measurable adhesive strength on hard tissues.

The fibrin adhesive strength values reported are limited to the commercially available Tisseel formulation and compared with what has been reported for the same material reported in other test models.

The metaphyseal region of the femur was selected, rather than the epiphysis or diaphysis, as this was a bone region that contained cancellous and cortical bone, within close proximity to osteochondral bone. The present test model is a more realistic representation of the mixed loading that occurs during physiological weight bearing, compared to the more common, simple push-out or pull-out test [26–29].

## Results

### Surgical approach & experimental design

**Fig. 1:**
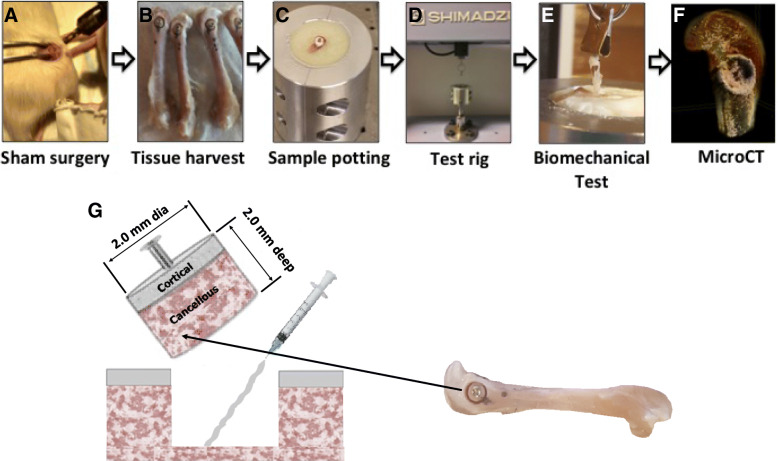
Surgical approach and experimental overview. The anatomical location of the defect and theoretical surgical approach is shown in a sham surgery (**a**, note that no actual surgeries were performed in the present study), whole femurs excised with defects clearly marked (**b**), after the fragment is reattached the femur is truncated to fit into the potting (**c**) and test rig (**d**), a tensile load is applied (**e**, Tisseel sample at failure point) and, after testing, a three dimensional reconstruction of each sample was used to analyze the bond thickness, adhesive failure mode and variability in tissue architecture (**f**, OsStic sample after failure). For clarity the dimensions of the excised/reattached fragment, and anatomical location are indicated (**g**)

A representative overview of the surgical approach and mechanical test process is shown in Fig. 1a-f. After a screw was inserted into an excised metaphyseal plug, the plug was removed and either OsStic or Tisseel was delivered by syringe, before the plug was reinserted (Fig. 1g) and the adhesive was allowed to cure. Markings were made (Fig. 1b) to ensure the plug was reinserted in the correct orientation. A tensile load was applied to the adhesive interface, between deep cancellous bone surfaces of the metaphyseal plugs.

### Potting epoxy exotherm

**Fig. 2:**
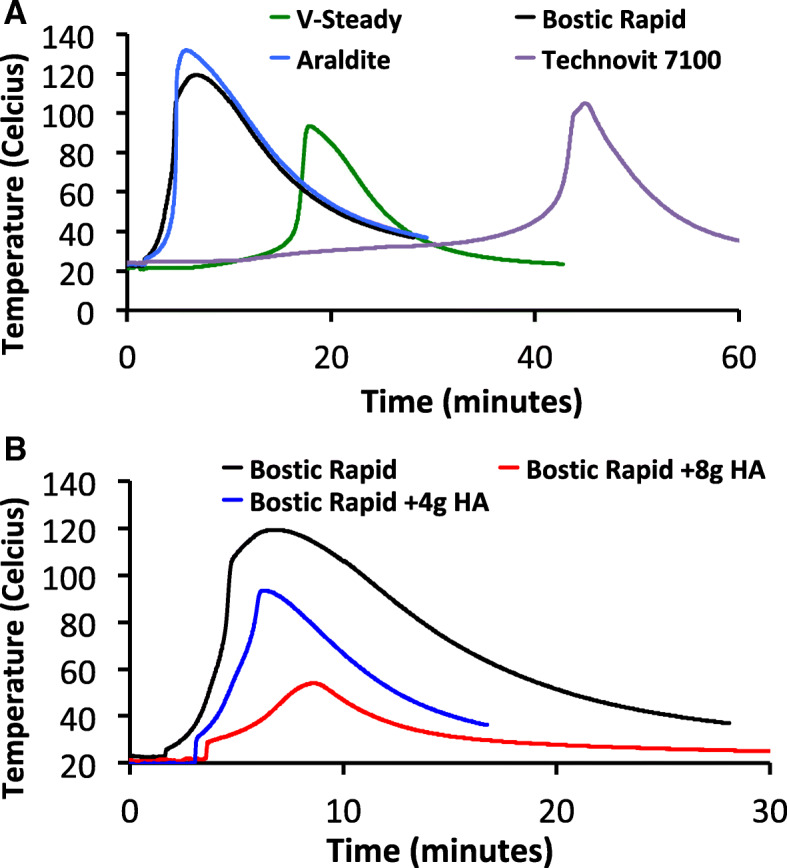
Exotherms of potting compounds and epoxies. The total heat release of three potting compounds, Technovit 7100, Araldite, and Bostic Rapid were compared to a clinical grade polymethylmethacrylate (PMMA), V-Steady (**a**). Since each material produced excessive heat while curing, calcium phosphate was added to reduce the heat release (**b**). An equal mixture, by weight, was found to minimize heat without noticeably impairing the potting strength

A rapidly polymerizing epoxy was needed, to minimize the effects of tissue decomposition (aging) on the mechanical test results. However, when 3 commonly used epoxies were screened, a sharp increase in the temperature of all potting compounds, associated with the exothermic process of rapid polymerization, was observed [28, 33]. All compounds produced exothermic temperatures that damaged the mounted tissue (Fig. 2a). In some cases, fluid was observed boiling out of the truncated portion of the diaphysis. A solution was devised that reduced the peak exotherm temperature without noticeably impairing the potting strength (Fig. 2b). Calcium phosphate powder was added to Bostic Rapid during curing, at varied weight percentages (wt%), and the peak curing temperature decreased from 120 degrees Centigrade (°C) to < 55 °C degrees. Damage to tissues from excessive polymerization temperatures can significantly impact biomechanical testing. This report represents the first published account of a lower temperature, rapidly polymerizing material for mechanical testing of tissues.

### Mechanical testing

**Fig. 3:**
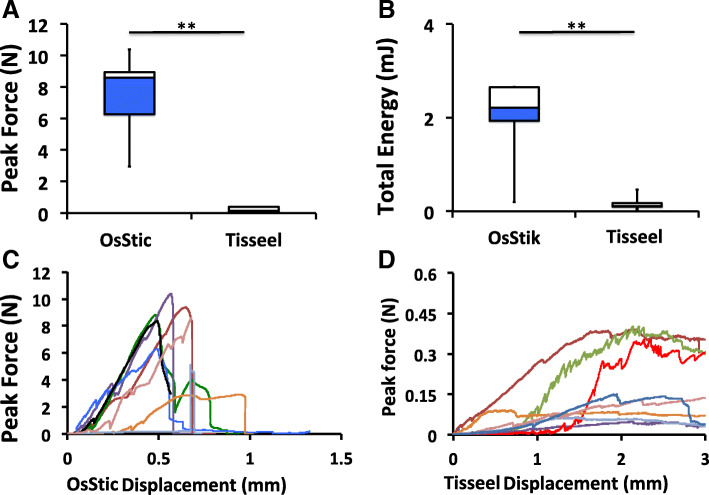
Adhesive bond strength of OsStic or Tisseel to metaphyseal bone. Box plots of failure strength of OsStic or Tisseel, bonded to metaphyseal bone, following pull-out testing (**a**). The average total energy (force integrated over displacement), for each sample at failure, is shown in (**b**). The force/displacement curve of each individual sample are shown for OsStic (**c**) or Tisseel (**d**). Note that the true bond thickness, and true strain values, could only be estimated from micro Computed Tomography (microCT) images after testing. Therefore, absolute forces and displacement values were used to produce force/displacement curves, rather than stress/strain curves. In Fig. 3a and b white and blue regions represent failure strength, and total energy, of samples in the 75th and 25th percentile, while whiskers represent the highest and lowest values in each group)

The average peak force at failure is shown in Fig. 3a. The adhesive interface encountered a mixed type of loading, with tensile force acting on the bottom (medial) cancellous surface, and shear force acting on the cancellous and cortical side surfaces of the metaphyseal plug. The average failure force of OsStic treated rodent bone was 36-fold stronger than with Tisseel (7.64 ± 2.39 N vs. 0.21 ± 0.16 N), after curing for 4 h. When the total energy was calculated, by integrating the force over total displacement (Fig. 3b), OsStic was 11-fold greater than Tisseel (2.06 ± 0.89 millijoule (mJ) vs. 0.17 ± 0.14mJ). Note that one sample in the OsStic group suffered a sharp, brittle failure, after minimal displacement. This sample resulted in a lower total energy, as seen by the long whisker (Fig. 3b).

The failure mode of the two adhesives differed, with OsStic producing sharp brittle failure while Tisseel samples remained weakly attached over long displacements. The force/displacement curves for each test sample are shown in Fig. 3c (OsStic) and Fig. 3d (Tisseel). Most importantly, the current test model produced measurable forces from murine bone, with large enough precision (35% Relative Standard Deviation (RSD)) that the failure strength of disparate materials was easily distinguished.

### Microcomputed tomography (MicroCT) analysis

**Fig. 4:**
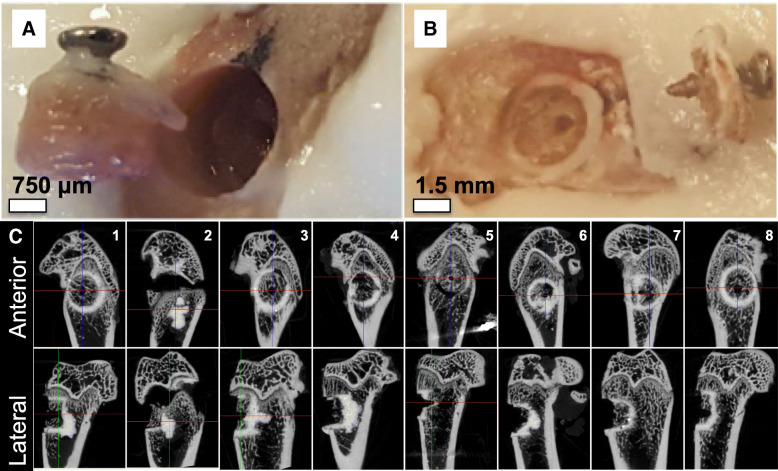
Failure analysis of reconstructed metaphyseal bone. The different type of failure is shown for Tisseel (**b**) and OsStic (**a**). In all Tisseel samples failure clearly occurred at the Tisseel/tissue interface (adhesive failure) and the entire plug, including the Tisseel material, was pulled out completely. In 6/8 OsStic samples failure occurred in cancellous bone, rather than within OsStic (cohesive failure) or at the OsStic/tissue interface (adhesive failure) (**c**)

The failure mode of Tisseel and OsStic are shown in Fig. 4a and b, respectively. Tisseel was pulled completely out of the defect site, failing adhesively (at the Tisseel/tissue interface) rather than cohesively (within the Tisseel material) (Fig. 4a). OsStic did not fail adhesively or cohesively in the present study; instead the surrounding cancellous tissue appeared to fail. In Fig. 4b the metaphyseal plug has fractured, leaving a cancellous portion glued to the defect site by OsStic, and the remaining (cortical and cancellous) portion of the plug attached to the screw. In 6 out of 8 samples, fractions of cancellous tissue from the displaced metaphyseal plug remained glued at the adhesive interface by OsStic (Fig. 4c, lateral view) the adhesive strength exceeding the fracture strength of murine cancellous bone.

## Discussion

The development of an animal model where there is no existing predicate is challenging and took several iterations before the present method produced results that were sufficient for evaluating adhesives, in bonding cancellous bone. The variables that affect the present results include surgical technique, intra-species differences, and those inherent to handling and applying any adhesive onto freshly fractured tissue surfaces of miniscule size. Nevertheless, the present results are accurate, reproducible (35% RSD), and precise enough to detect differences with statistical analysis. The observed bond strengths (0.21 N per 0.157 cm squared (cm^2^), or 0.013 MPa for Tisseel) in the present study closely match published values in other ex vivo test models [11], and are slightly lower than reported values in vivo [13, 34, 35]. The difference in adhesive strength, between published values for Tisseel in vivo and the present study, likely arises from osteoid deposition and tissue healing, which augment the bond strength of reconstructed fracture surfaces in vivo*.* Collectively, these results suggest that the present model may accurately predict performance in vivo (model accuracy).

The rodent metaphyseal defect model, presented herein, includes a number of potential limitations. First, the bond thickness varied considerably between samples, likely due to the subjective process of applying an “appropriate” force, by the operator, during repositioning/fixation of the metaphyseal fragment. Counter-intuitively, this is actually an advantage rather than a limitation, for the present model. Slight variations in force, which also occur in the clinic during surgery, will produce varied adhesive bond thickness and strengths. Some samples will cure with overly thick or thin bonds. Therefore, the most clinically relevant metric of the present study, after model accuracy, may be whether the test model is sensitive enough to confidently identify the lowest range of adhesive strength for each test group (i.e., can one distinguish the weakest quartile of samples for each group, and whether samples in *this quartile* still produces sufficient strength for clinical application). For both test groups, peak force values in the lowest quartile were within 2-sigma from the group average.

A second limitation of the present study was the use of microCT imaging only after mechanical testing each sample. MicroCT imaging is necessary to define multiple parameters that are critical for any hard tissue adhesive: the average bond thickness; and whether the adhesive was evenly distributed over the entire tissue interface or was spread unevenly, and whether the plug was repositioned unevenly. These factors will determine whether the results are inaccurate due to stress concentrations (i.e. premature failure, or overestimation of adhesive strength). In this study microCT imaging was used only after mechanical testing to prevent tissue deterioration arising from evaporation during the scanning. For future testing, a procedure for imaging before and after mechanical testing must be validated to ensure the process does not affect the mechanical test results. The current model should also be extended, in future testing, to epiphyseal tissue, specifically for osteochondral tissue reconstruction.

Finally, this is the only reported rodent model that can evaluate adhesion strength of bone fragments (bone to bone adhesion in a pullout test), in load bearing cancellous tissue, adjacent to a joint. As a novel test model further validation is needed to verify the sensitivity and accuracy of the model for other adhesive materials, particularly materials where the expected adhesive strengths, and material properties, are less disparate.

The least expected development in the present study was the need for a potting material that did not damage the tissue. It was expected that bone tissue potting agents described in the scientific literature would not produce excessive exothermic curing temperatures. However, none of the commonly reported potting materials were found, in the present study, to be appropriate for potting whole tissues for biomechanical testing. This study represents the first published report of a potting material composition that is suitable for rapid biomechanical testing of small bone tissue samples.

## Conclusions

The present biomechanical test model represents the first report of an ex vivo model using murine bone that is sensitive enough to detect bone tissue adhesive strength, between two different types of tissue adhesives. This model has the potential to accelerate the evaluation process for calcified tissue adhesives, while avoiding the significant costs associated with large-animal studies during the pre-clinical evaluation stage.

## Methods

### Materials

Tisseel fibrin adhesive was produced by Baxter (Baxter Medical AB, Kista, Sweden). OsStic was created by mixing commercially available phosphoserine (Flamma, S.p.A. Italy) and alpha tricalcium phosphate powders (Robert Mathys Stiftung foundation, > 97% pure), at a 3:7 M ratio, with a liquid to powder ratio of 0.2 ml per gram (ml g^− 1^) and deionized water as the liquid [17]. Murine femurs were isolated from sacrificed Sprague Dawley rats, wrapped in gauze, soaked in phosphate buffer saline (PBS) and stored at -20 °C. Prior to testing the femurs were stored at 4 °C and allowed to thaw for 24 h, before warming in phosphate buffered saline, at 37 °C. Although fibrin glue is indicated for soft tissue and non-load bearing applications, Tisseel was selected as a control material because it produces measurable adhesive force in calcified tissues [13, 18], and has been used as a basis for comparison for orthopaedic applications [13, 36]. Bostic Rapid and Araldite were purchased from Bauhaus AB (Uppsala, Sweden). Technovit 7100 was purchased from Histolab (Gothenburg, Sweden). V-Steady was generously provided by G21 S.r.l. (Italy). All epoxies were prepared as suggested by the manufacturer. Calcium phosphate (hydroxyapatite, 90%) powder was purchased from Sigma Aldrich.

### Surgical model

Rat femurs were collected from euthanised rats that had been used in a previous study with the ethical number (5.8.18–11,832/2017.). The femurs were dissected out and the surgical site was selected by identifying the lateral epicondyle. A metaphyseal fragment was created by drilling with an irrigated trephine (outer Ø2.8 mm, inner Ø2.0 mm, Dental mind, Gothenburg, Sweden), to an approximate depth of 2 mm. A pilot hole was drilled with a Ø0.80 mm drill bit (Cousins Material House Ltd. UK) and a steel screw (Micron Wings Screws Self Tapping Ø1.0 mm × 3 mm Pan Head 304 Stainless Steel) was manually inserted using a pin-vice/screw holder (Cousins Material House Ltd. UK). After inserting the screw to a depth of 2 mm, the osseous fragment was gently displaced by applying a slight bending force to the screw. The fragment was removed and an approximate volume of 0.2 ml was injected into to the defect site, either through the custom syringe provided as part of the Tisseel kit, or through a 1cm^3^ (cc) syringe for OsStic. Delivery was completed within 30 s and the osseous fragment was repositioned, relative to orientation markings that were placed on either side of the drill site, into the metaphyseal defect. The fragment was held in place for 5 min before moving the entire femur to phosphate buffered saline, at 37 °C for 1 h.

### Mechanical testing and sample potting

During potting, rodent femurs were oriented with a screw inserted into the metaphyseal plug, perpendicular to the potting surface. The femur was truncated with a diamond bladed bandsaw (IMEB Inc., USA) in the diaphyseal region, to fit the size of the potting mold, and was potted as a 50% (wt.%) mixture of Bostic Rapid epoxy and calcium phosphate (8-g (g) epoxy and 8 g of calcium phosphate). The epoxy was allowed to cure for 4 h at 37 °C, in humidity, throughout the curing process. The exotherm of the epoxy was monitored with a thermocouple (Omega JMTSS M050G-150) and temperature input module (National Instrument NI9211), with measurements taken every second, to ensure the curing temperature remained below 55 °C. The curing temperature was monitored when different amounts of epoxy were replaced with calcium phosphates, in 4 g increments. The following compositions were tested: 16 g of epoxy; 12 g of epoxy with 4 g of calcium phosphate; 8 g of epoxy with 8 g of calcium phosphate.

Approximately 16 g of potting epoxy was prepared for each femur, in an 18 cc cup (Ø 3.8 cm (cm)). A custom designed clamp was placed under the screw and preloaded to 0.5 N, as part of a tensile testing rig show in Fig. 1d. The screw and osseous fragment were then displaced (tensile loading) at rate of 1 mm/min on a Shimadzu AGS-X mechanical testing machine, equipped with a 50 N load cell (#SM-50 N-168, Shimadzu Europa). Force-displacement curves were generated with software provided by Shimadzu (Trapezium-X Lite version 1.01, Shimadzu Europa). The total energy until failure was calculated by integrating the force for each unit of displacement (zero displacement occurred when the detected force exceeded 0.01 N), using Origin software. For Tisseel samples the force was integrated to a maximum displacement of 2 mm, at which point the plug was completely displaced from the defect site.

### Microcomputed tomography (microCT) imaging

MicroCT imaging was performed on a Skyscan 1176 (Bruker MicroCT, Kontich Belgium), with 50 kV (kV) voltage, 500 mA current, 0.5 mm Al filter, average 4 and 360° scan with a voxel size of 18 μm (μm), images were reconstructed using NRecon, and visualized using CTVox software (both from Bruker MicroCT, Kontich Belgium). To avoid deterioration of the mechanical properties of biological samples (i.e. drying out of tissue, etc.) or the adhesive, from prolonged imaging, MicroCT was performed only after biomechanical testing. The defect/plug dimensions, and approximate adhesive bond thickness were calculated by taking the average measured values using CTVox software (three measurements for defect/plug size, two measurements for bond thickness).

### Statistics

The force and energy values were compared between the two adhesives using a two-tailed Student’s t-test, with significance set for *p* < 0.05 (indicated with * for *p* < 0.05 and ** for *p* < 0.01). Each group contained 8 samples.

## Abbreviations

cc: Cubic centimetre
cm: Centimeter
cm^2^: Centimetre squared
FDA: Food and Drug Administration
g: Gram
KPa: Kilopascal
kV: Kilovolt
MDR: Medical Device Reporting
microCT: Micro Computed Tomography
mJ: Millijoule
ml: Millilitre
ml g^− 1^: Millilitre per gram
mm: Millimetres
MPa: MegaPascal
N: Newtons
Ø: Diameter
^o^C: Degrees centigrade
PBS: Phosphate buffer saline
PMMA: Polymethylmethacrylate
The 3R’s: The principles of Replacement, Reduction and Refinement
wt.%: Weight percentages
μm: Micrometre

## References

[1] Amin S, Achenbach SJ, Atkinson EJ, Khosla S, Melton LJ (2014). Trends in fracture incidence: a population-based study over 20 years. J Bone Miner Res.

[2] Sozen T, Ozisik L, Basaran NC (2017). An overview and management of osteoporosis. Eur J Rheumatol.

[3] van Staa TP, Dennison EM, Leufkens HG, Cooper C (2001). Epidemiology of fractures in England and Wales. Bone..

[4] Kratzel C, Bergmann C, Duda G, Greiner S, Schmidmaier G, Wildemann B (2008). Characterization of a rat osteotomy model with impaired healing. BMC Musculoskelet Disord.

[5] Kuhle J, Angele P, Balcarek P, Eichinger M, Feucht M, Haasper C (2013). Treatment of osteochondral fractures of the knee: a meta-analysis of available scientific evidence. Int Orthop.

[6] Ng WM, Al-Fayyadh MZM, Kho J, Seow Hui T, Mohamed Ali MRB (2017). Crossing suture technique for the osteochondral fractures repair of patella. Arthrosc Tech.

[7] Heiss C, Kraus R, Schluckebier D, Stiller A-C, Wenisch S, Schnettler R (2006). Bone adhesives in trauma and orthopedic surgery. Eur J Trauma.

[8] Schreader KJ, Bayer IS, Milner DJ, Loth E, Jasiuk I (2012). A polyurethane-based nanocomposite biocompatible bone adhesive. J Appl Polym Sci.

[9] Farrar DF (2012). Bone adhesives for trauma surgery: a review of challenges and developments. Int J Adhes Adhes.

[10] Cedano Serrano FJ, Pinzón LM, Narváez DM, Castro Paéz CI, Moreno-Serrano CL, Tabima DM (2017). Evaluation of a water-resistant and biocompatible adhesive with potential use in bone fractures. J Adhes Sci Technol.

[11] Dehne T, Zehbe R, Kruger JP, Petrova A, Valbuena R, Sittinger M (2012). A method to screen and evaluate tissue adhesives for joint repair applications. BMC Musculoskelet Disord.

[12] Kandalam U, Bouvier AJ, Casas SB, Smith RL, Gallego AM, Rothrock JK (2013). Novel bone adhesives: a comparison of bond strengths in vitro. Int J Oral Maxillofac Surg.

[13] Keller J, Andreassen TT, Joyce F, Knudsen VE, Jorgensen PH, Lucht U (1985). Fixation of osteochondral fractures. Fibrin sealant tested in dogs. Acta Orthop Scand.

[14] Granskog V, García-Gallego S, von Kieseritzky J, Rosendahl J, Stenlund P, Zhang Y (2018). High-performance thiol–Ene composites unveil a new era of adhesives suited for bone repair. Adv Funct Mater.

[15] Grover LM, Gbureck U, Farrar D, Barralet JE (2006). Adhesion of a novel calcium phosphate cement to cortical bone and several common biomaterials. Key Eng Mater.

[16] Kirillova A, Kelly C, Windheim N, Gall K (2018). Bioinspired mineral–organic Bioresorbable bone adhesive. Adv Healthc Mater.

[17] Pujari-Palmer M, Guo H, Wenner D, Autefage H, Spicer CD, Stevens MM, et al. A novel class of Injectable bioceramics that glue tissues and biomaterials. Materials. 2018;11(12). 10.3390/ma11122492 PMC6316977 30544596

[18] Hoffmann B, Volkmer E, Kokott A, Augat P, Ohnmacht M, Sedlmayr N (2009). Characterisation of a new bioadhesive system based on polysaccharides with the potential to be used as bone glue. J Mater Sci Mater Med.

[19] Shao H, Bachus KN, Stewart RJ (2009). A water-borne adhesive modeled after the sandcastle glue of P. Californica. Macromol Biosci.

[20] Nyarko A, Barton H, Dhinojwala A (2016). Scaling down for a broader understanding of underwater adhesives – a case for the Caulobacter crescentus holdfast. Soft Matter.

[21] Lim J, Jariwala A, Wigderowitz C, Drew T. The use of bone adhesive for fracture fixation in long bones- a biomechanical study. Front Biol Life Sci. 2014;2(2):29–33.

[22] Le TT, Vo HV, Webb LX (2016). Investigation of kryptonite™ bone cement in hybrid screw configurations of locking plate humeral midshaft fixation: a study of surrogate bone model. J Orthop.

[23] Ball CG, Grondin S, Pasieka J, Kirkpatrick A, MacLean A, Cantle P, Dixon E, Schneider P, Hamilton M (2018). Examples of dramatic failures and their effectiveness in modern surgical disciplines: can we learn from our mistakes?. J Comp Eff Res.

[24] Shah NV, Meislin R (2013). Current state and use of biological adhesives in orthopedic surgery. Orthopedics (Healio).

[25] Clemens N (2017). The new European medical device regulation 2017/745: Main changes and challenges. Associate of clinical research professionals.

[26] Bhagat V, O’Brien E, Zhou J, Becker ML (2016). Caddisfly inspired phosphorylated poly (ester urea)-based degradable bone adhesives. Biomacromolecules..

[27] Berzins A, Shah B, Weinans H, Sumner DR (1997). Nondestructive measurements of implant–bone interface shear modulus and effects of implant geometry in pull-out tests. J Biomed Mater Res.

[28] Bell S, Ajami E, Davies JE. An improved mechanical testing method to assess bone-implant anchorage. J Vis Exp. 2014;(84):e51221. 10.3791/51221 PMC4122136 24561765

[29] Seong W-J, Grami S, Jeong SC, Conrad HJ, Hodges JS (2011). Comparison of push-in versus pull-out tests on bone-implant interfaces of rabbit tibia dental implant healing model. Clin Implant Dent Relat Res.

[30] Sidle DM, Maas CS (2008). Determination of shear strength of periosteum attached to bone with bioglue surgical adhesive. Arch Facial Plast Surg.

[31] Manikandan ATK, Thiruselvi T, Gnanamani A. Engineered protein adhesive gel as an Osteo conductive material for bone healing. Juniper Online J Mater Sci. 2017;2(2):7. Article ID 236231.

[32] Krticka M, Michlovska L, Nekuda V, Chamradova I, Sojka K, Kaiser J (2018). Shear compression and three-point bending force testing on ex vivo model of fractured pig femur fixed with novel biodegradable injectable polymer composite glue. Orthop Proc.

[33] Rice CA, Riehl J, Broman K, Soukup JW, Gengler WR (2012). Comparing the degree of exothermic polymerization in commonly used acrylic and provisional Composite resins for intraoral appliances. J Vet Dent.

[34] Chivers RA, Wolowacz RG (1997). The strength of adhesive-bonded tissue joints. Int J Adhes Adhes.

[35] Mo X, Iwata H, Ikada Y (2010). A tissue adhesives evaluated in vitro and in vivo analysis. J Biomed Mater Res A.

[36] Kaplonyi G, Zimmerman I, Frenyo AD, Farkas T, Nemes G (1988). The use of fibrin adhesive in the repair of chondral and osteochondral injuries. Injury..

